# Differential Gene Expression of Innate Immune Response Genes Consequent to *Solenopsis invicta* Virus-3 Infection

**DOI:** 10.3390/genes14010188

**Published:** 2023-01-10

**Authors:** V. Renee Holmes, J. Spencer Johnston

**Affiliations:** Department of Entomology, Minnie Bell Heep Center, Texas A&M University, Suite 412 2475 TAMU, 370 Olsen Blvd, College Station, TX 77843, USA

**Keywords:** transcriptome, innate immune pathways, Warburg effect, fire ant control, pathogenesis

## Abstract

The red imported fire ant *Solenopsis invicta* Buren (fire ant hereafter) is a global pest that inflicts billions of dollars in damages to the United States economy and poses a major threat on a global scale. Concerns with the broad-spectrum application of insecticides have facilitated the hunt for natural enemy-mediated controls. One of these, the virus *Solenopsis invicta* virus-3 (SINV-3 hereafter) is exceptionally virulent in laboratory settings. However, despite high mortality rates in the laboratory and documented widespread SINV-3 prevalence in the southern United States, the fire ant remains a major pest. To explore this paradox, we document the immune response elicited by the fire ant when infected with SINV-3. We sequence the fire ant transcriptome prior to and following infection with SINV-3, and identify and discuss in detail genes in immune response pathways differentially expressed following infection with SINV-3. This information provides insights into genes and pathways involved in the SINV-3 infection response in the fire ant and offers avenues to pursue, to suppress key immune response genes and force the fire ant to succumb to SINV-3 infection in the field.

## 1. Introduction

Since its introduction decades ago, the fire ant *S. invicta* Buren has become one of the most widely recognized and detested insect pests in the southern United States, and, within the last decade, has become a global pest [1]. As is the case with many imported ant species, the fire ant displays exceptional aptitude for colonizing non-native ranges following introduction [2,3]. The fact that they are not considered a major pest in their native habitat [3,4] is important information and likely due in part to the natural enemies they escape in the invasive range. While chemical insecticides are effective for fire ant control [5], their application can raise concerns with environmental contamination and off-target effects [6], and can be problematic for large-scale application [7,8]. For this reason, the use of natural enemies in fire ant control has long been an area of interest. Among natural enemies are an increasing number of viral pathogens, of which SINV-3 appears to hold promise, creating disease outcomes in the fire ant reminiscent of colony collapse disorder (CCD) in the honey bee *Apis mellifera* [9,10,11].

The disease landscape of SINV-3 and its interaction with the fire ant host appears to be complex; the fire ant’s invasive range continues to expand despite widespread prevalence of SINV-3, albeit at low levels [12]. One of the major aspects of the fire ant SINV-3 disease’s natural history that remains to be studied is the immune response mounted by the fire ant to combat SINV-3 infection. Eusocial insects face a unique disease landscape compared to their solitary invertebrate counterparts. Nestmate proximity, food sharing via trophallaxis, and close relatedness of nestmates provide an environment especially susceptible to epidemic disease outcomes [10,13]. Further, the insect immune response is limited when compared with the intricate antibody-mediated adaptive immune systems seen in vertebrates. In the vertebrate system, immune challenge is mediated by both adaptive and innate systems, whereas the insect system is controlled entirely by innate immune response pathways. Of these, the immune deficiency (Imd), Toll, RNAi, janus kinase (jnk), glycolysis and apoptosis pathways are implicated in the viral immune response of other eusocial Hymenopterans and the model organism Drosophila [10,14,15,16].

Several characteristics of the fire ant make it a prime candidate for control via viral natural enemies. Along with the rudimentary immune systems of insects, nest size and connectedness has been shown to be positively correlated with an increase in the prevalence of viral pathogens [17]. Laboratory studies have also shown that the pathogen SINV-3 progresses with catastrophic outcomes, including death of brood, reduction in foraging behavior and reduction of queen fecundity [11]. Further, SINV-3 has been documented in invasive ranges in both Florida [18,19] and Texas [12], so it is logical that the fire ants in these areas should be suffering the consequences of SINV-3 infection and populations declining as a result. This is not the case, however; fire ants continue to thrive in these areas. All of this considered, we hypothesize that the fire ants continue to thrive because the fire ant immune system can respond successfully to SINV-3 infection. To test that hypothesis, we studied the fire ant innate immune response to SINV-3 infection.

We addressed the question of red imported fire ant immune responses to SINV-3 using a transcriptome sequencing approach. We first established feral fire ant colonies in the lab. After confirmation that the colonies were of the polygyne social form and free of infection with SINV-3 and other known fire ant pathogens, we sequenced the transcriptome of each colony. We then infected each sampled colony with SINV-3 and repeated the sequencing step. Successive sequencing revealed differential gene expression in several known immune response pathway components. Among the insights gathered from this work is the potential for novel RNA interference approaches to suppress the fire ant immune system and increase the deleterious outcomes caused by SINV-3.

## 2. Methods

### 2.1. Field Collection of Fire Ants

Six feral fire ant colonies were collected from the Millican Reserve wildlife conservancy in College Station, TX, USA, an area heavily infested with fire ants and minimally disturbed by human activity. Each mound collected for use in the study was large and easily visible, with accessible brood and multiple visible queens at the time of collection. Once colonies to be included in the study were located, each was dug up using a shovel and placed in separate 10-gallon buckets for transportation to the laboratory.

### 2.2. Laboratory Colony Establishment

Following field collection, each colony was separated from the mound substrate using the drip method [20]. Before inclusion in the study, social form and health status were evaluated. To identify social form, DNA was extracted from 10 pooled workers using a DNeasy Blood and Tissue Kit (Qiagen, Germantown, MD, USA) and amplified via PCR, using multiplex primer sets for the Gp-9 locus. Each colony included in this study was confirmed to be polygyne by the presence of one 517 bp amplicon (Gp-9 *^B^*) and one 423 bp amplicon (Gp-9 *^b^*), using gel electrophoresis [21]. Another ten pooled workers were collected from each colony, extracted for total RNA (Trizol) [22] and quantitative PCR (qPCR) confirmed the sample to be free from SINV-3 infection using primers specific to SINV-3 RNA-dependent RNA polymerase (RdRp) (5′-AAGGAGTTTGTGTATTAGTTGCAATGCCAGAATCT-3′; 5′-CTGCTGGTATGATGGCAACAGATCCTTCTGT-3′) [19,23].

After determination of social form and confirmation of a SINV-3 negative status, each colony was placed in a sweater box lined with fluon to prevent the ants from escaping. Each sweater box contained black painted petri dishes for the ants to use as rearing chambers for brood and queens. Each colony was allowed ten days to acclimate to the laboratory environment, during which time they were allowed free choice access to purified water and an artificial fire ant diet [24].

### 2.3. Infection Inoculum Preparation

Infective inoculum with SINV-3 was prepared using an artificial fire ant diet [24] with the addition of 500 milligrams of prepared infected fire ant workers to form an inoculum slurry. Ants used in the inoculum preparation were collected in the field and identified as infected via qPCR using RdRp-specific primers (methods Section 2.2). The infected ants used in the infection inoculum were ground into a fine paste using Invitrogen sterile mortar and pestles and filtered through a 20 nm nylon mesh (Spectra/Mesh), to remove sclerotized fire ant body parts. A sample of homogenized ant paste was extracted for total RNA using Trizol [22] and the SINV-3 titer was measured using qPCR to determine copy numbers of SINV-3 present in inoculum. The ant paste infection inoculum yielded 10^8^ genome equivalents/mL when tested. Filtered ant paste was mixed into the prepared artificial fire ant diet to form an infective slurry, 1 mL of which was then aliquoted into 1.5 mL microfuge tubes to be fed to ants in the infection study (Section 2.5).

### 2.4. Sample Preparation of Uninfected Ants

Following field collection and establishment (methods Section 2.2), 10 major workers were collected from each colony. Sampled workers were pooled and extracted for total RNA using Trizol [22]. Extracted RNA concentration was measured using nanodrop and confirmed to have a minimum of 150 ng/μL total RNA. Uninfected samples were used as controls to document baseline gene expression prior to infection. Total RNAs were sequenced by Novogene laboratories (Davis, CA, USA).

### 2.5. Infection Methodology and Infected RNA Sample Preparation

After control samples were collected from each colony, study colonies were subjected to SINV-3 infection. One 1.5 mL microfuge tube containing the infective diet aliquot was placed in each of the experimental colonies, and ants were allowed to feed ad libitum until the contents of the tube were emptied (around 4 days). After allowing each colony to feed on the infective inoculum until contents were emptied, each colony was sampled by collecting 10 major workers at 5 days post-exposure to infection inoculum. Sampled ants were then extracted for total RNA (Trizol [22]) and subjected to qPCR (see methods Section 2.2 for primer sequences) to confirm replicating SINV-3 infection. Extracted RNA samples were then subjected to nanodrop and confirmed to contain a minimum concentration of 150 ng/μL total RNA. Infected samples were then sent to Novogene laboratories (Davis, CA, USA) for sequencing. Differential gene expression between the control uninfected status and infected status was quantified.

### 2.6. RNA Preparation and mRNA Sequencing

Illumina sequencing was performed by Novogene laboratories (Davis, CA, USA). Each RNA sample contained a minimum concentration of 150 ng/mL RNA. All samples were subjected to QC (Agilent2100) and library preparation was performed using NEB Non-Directional Lib Prep. The workflow process proceeded with total RNA detection, ribosomal RNA removal, cDNA synthesis and purification cDNA repair alongside addition of adaptors, fragment selection and PCR enrichment, library quality assessment and Illumina sequencing. Total reads generated using Illumina sequencing and total reads mapped to the fire ant genome are found in Table 1. After reads were prepared for each sample, they were aligned to the genome of *S. invicta* using HISAT2 v2.0.5.

Analysis was then performed on the resulting sequenced reads against the *S. invicta* genome by showing the quality of raw sequence data, alignment of referenced sequences using the IGV software following gene expression analysis, RNA sequencing correlation analysis, and analysis and identification of differentially expressed genes (DEGs). Identification of genes was accomplished using DESeq2 and volcano plot; gene ontology and scatter plots of KEGG enrichment DEGs were performed using the Magic Novogene package. Genes were considered significantly differentially expressed when the adjusted *p*-value (padj) ≤ 0.05 and log2FoldChange > 1.

## 3. Results

### 3.1. Colony Level Outcomes

**Figure 1:**
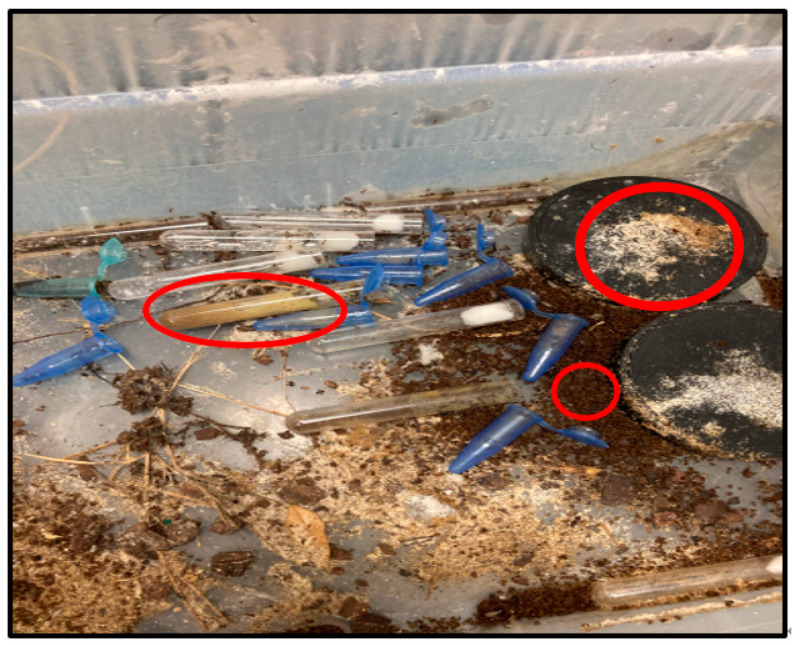
Image of one of the experimentally infected colonies at day 10 post-infection. Note the reduced feeding following infection marked by the full diet tube (far left red circle), large midden pile (small circle, middle right) and midden pile of deceased brood (far right).

Following colony field collection, all newly established laboratory colonies were allowed two weeks to become acclimated to laboratory conditions. Within a few days (~7), all colonies were producing brood, foraging, and maintaining productive queens, with no observable evidence of decline when free of SINV-3 infection. The colonies were then infected with SINV-3 as described above. Within a six-day period post exposure to SINV-3, brood volume was either substantially reduced or entirely deceased, with noticeable brood presence in the midden pile. Within a ten-day post exposure period, all study colonies had succumbed to infection *(*Figure 1). In all 6 inoculated colonies, decline of the colony proceeded similarly. In all cases, the infection outcome began with noticeable brood death; all or nearly all brood had died within a few (3–6) days. Within the same time frame or shortly thereafter, workers began to die. The death rate was rapid enough that remaining workers no longer moved the deceased to the midden pile. Deceased queens were also visible in the brood chambers.

### 3.2. Transcriptome Sequencing Results

#### 3.2.1. Differential Expression Following SINV-3 Exposure

**Figure 2:**
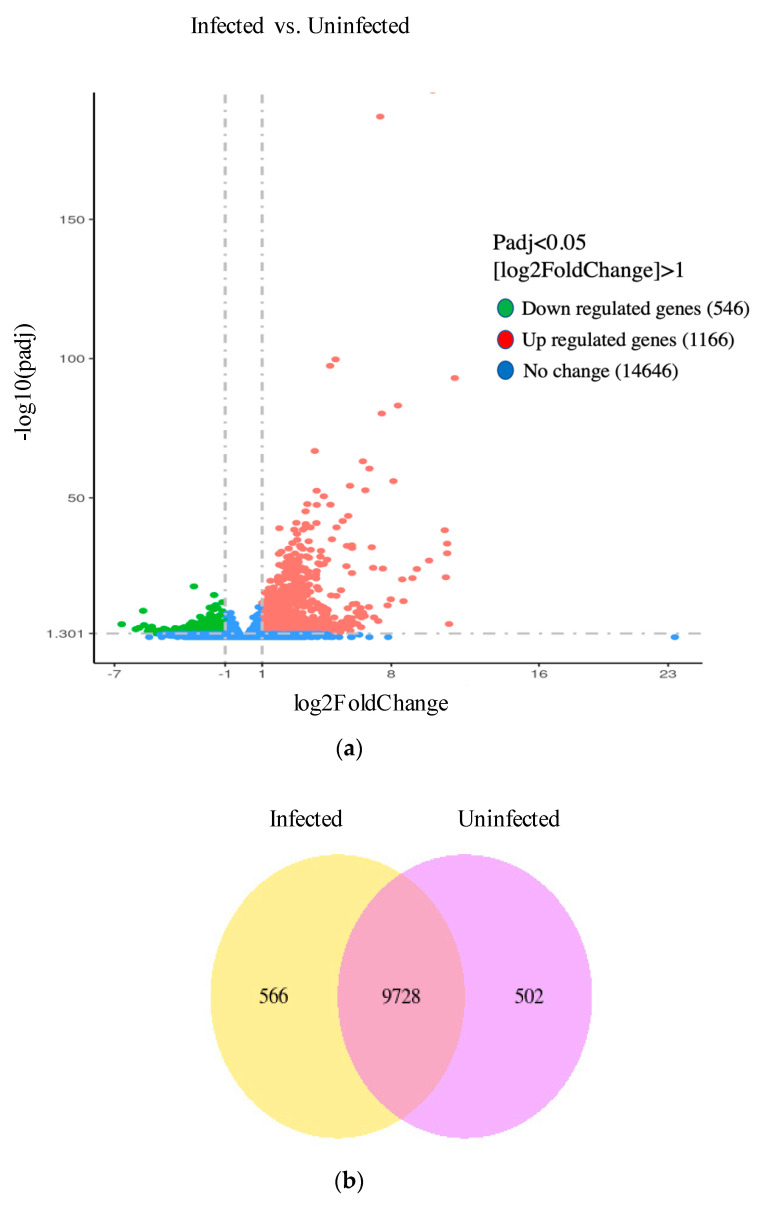
(**a**) Volcano plot showing differential expression (X-axis) across treatment and expression levels within groups (Y-axis), where (padj) is the adjusted *p*-value. (**b**) A total of 566 genes were uniquely expressed in infected colonies (yellow), 9728 were expressed in both infected and uninfected colonies (overlap), and 502 genes were uniquely detected in uninfected colonies.

Total reads generated from Illumina sequencing and total reads mapped to *S. invicta* genome can be found in Table 1. Significant levels of differential expression were seen when the transcriptomes of colonies prior to and following infection were compared. The total distribution of significantly differentially expressed genes are represented by the volcano plot (Figure 2a), where green dots represent downregulated genes, red dots represent upregulated genes, and blue dots represent genes not significantly differentially expressed. When the adjusted *p*-value (padj) was set to 0.0500 and log2FoldChange > 1, 546 genes were down regulated, and 1166 were upregulated. The 14646 genes that were not significantly differentially expressed were dropped from further consideration. A few genes (transcripts) were detected only when free of virus or only when exposed. A total of 566 genes were uniquely expressed at detectable levels in infected colonies, whereas 502 were expressed at detectable levels exclusively in uninfected colonies (Figure 2b). A total of 9728 genes were detected and scored in both infected and uninfected colonies. Most (~68%) of the genes that were significantly differentially expressed were upregulated, whereas comparatively few differentially expressed genes were downregulated (~32%) (X-axis of Figure 2a) and those were expressed in relatively low levels compared to the expression of upregulated genes (Y-axis of Figure 2a).

#### 3.2.2. Principal Component Analysis

**Figure 3:**
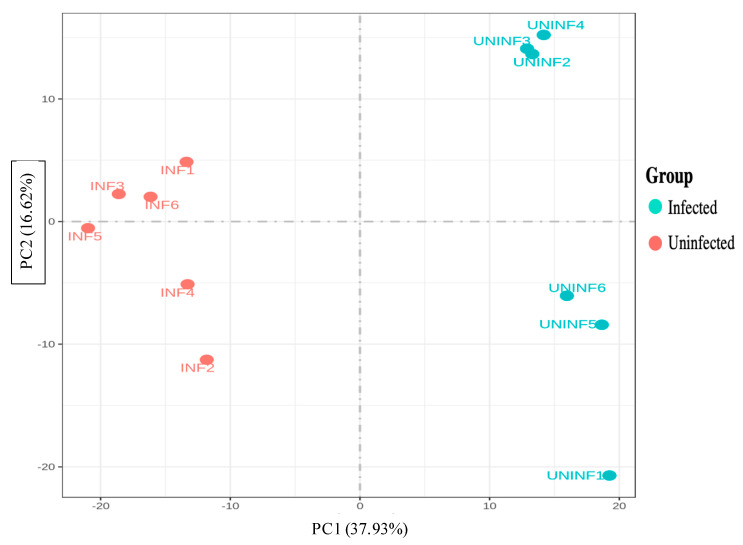
Principal component analysis. The very different expression levels between the infected and uninfected statuses are reflected on the PC1 axis. The PC2 axis reflects that variation among uninfected colony level expression is relatively wide, whereas variation in colonies infected with SINV-3 is much narrower.

A principal component plot (Figure 3) shows variation in expression between the two treatment groups. PC1 accounts for 37.93% of the variation and shows the substantial differential expression of infected versus uninfected fire ant colonies. PC2 accounts for an additional 16.62% of the variation. The variation accounted for by PC2 demonstrated high variation among uninfected colonies and low variation among infected colonies. The PC1/PC2 co-plot shows the clear separation between infected and uninfected colonies. Uninfected, wild-caught colony expression is highly variable, while infected colony expression patterns clustered tightly, regardless of PC2 expression levels prior to infection.

#### 3.2.3. Combined Heat Map of Differentially Expressed Genes between Infected and Uninfected Fire Ant Workers

**Figure 4:**
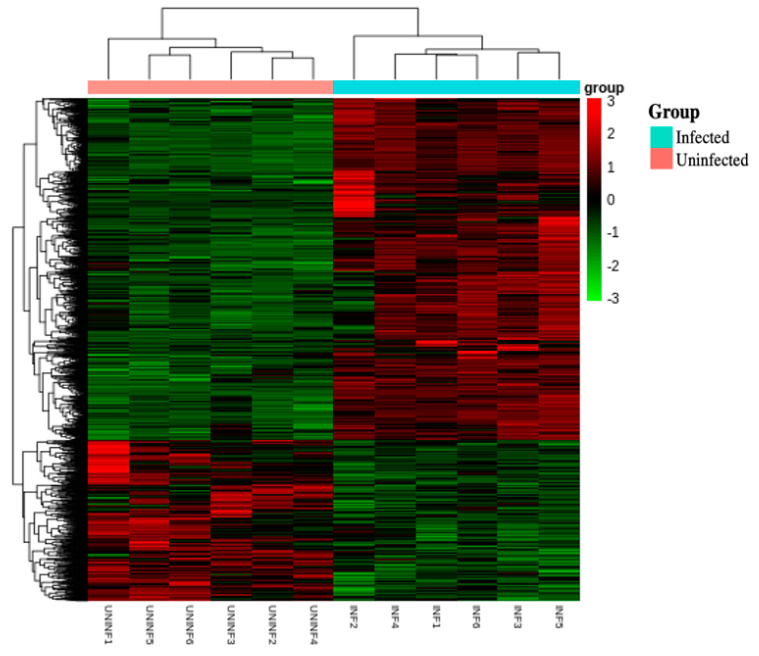
Combined heat map. Here, the red shade represents the more abundant gene expression in the corresponding sample, and green represents the less abundant gene expression.

The combined heat map (Figure 4) shows the phylogenetic relationship between infected and uninfected pooled fire ant colonies, as represented by the tree across the top of the heatmap. The tree shows that infection status caused divergence into two separate clades, infected and uninfected. Along the left border of the heat map is the phylogeny of gene expression levels, which separate into two major groupings. The top grouping shows those genes lowly expressed in the uninfected and highly expressed in the infected colonies. The lower grouping shows the opposite, with genes in this grouping being lowly expressed in infected colonies and highly expressed in uninfected colonies.

#### 3.2.4. Gene Ontology of Differentially Expressed Genes

**Figure 5:**
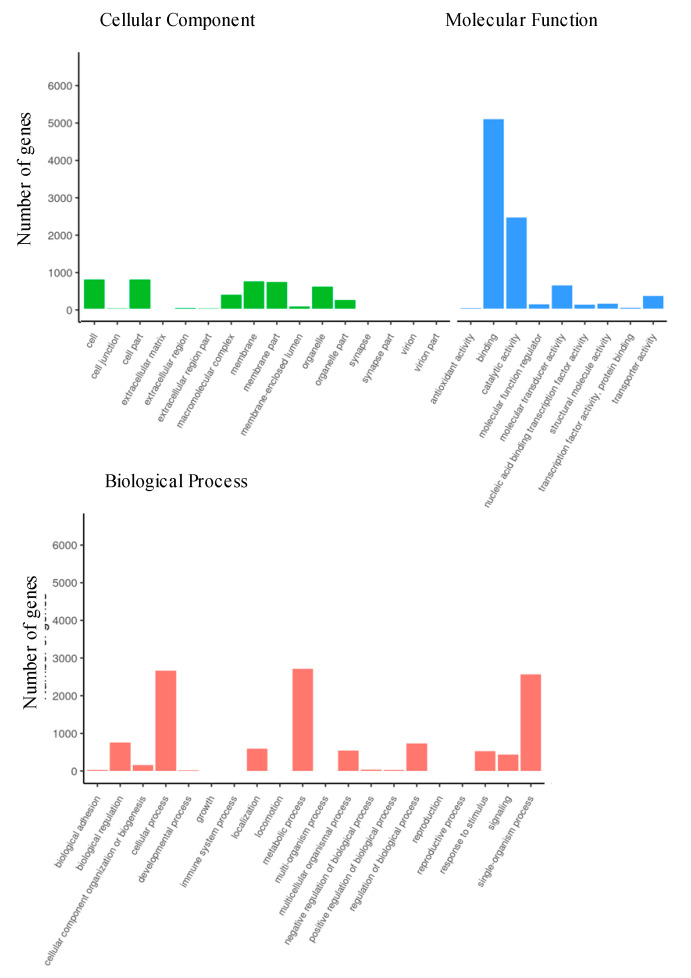
Gene ontology (GO) histograms showing GO enrichment of differentially expressed genes (DEGs) between infection status treatment groups, divided into categories: biological processes, cellular components, and molecular function. Green bars represent cellular components, blue represent molecular functions, and red represent biological processes.

Genes with orthologs to the honey bee genome were subjected to gene ontology analysis, which grouped DEGs into one of the three major categories: biological processes, cellular components, and molecular function (Figure 5). Within the category biological process, 85 differentially expressed genes were implicated in immune response, defense response, and cellular response to immune challenge. Exosome and vesicle formation genes were also differentially expressed (40 genes) as a part of molecular function, cellular components, and biological process classifications. Among the most relevant genes identified through gene ontology were system process genes, including Toll and Imd pathway components that are implicated in the invertebrate viral immune response. Within the cellular component function, 11 genes associated with cytoplasmic vesicle formation and 10 genes associated with membrane-bound vesicle formation were upregulated in infected ants. Within the molecular function subgroup, 79 genes were upregulated in infected ants. Of these, 20 upregulated genes were associated with positive regulation of the immune response, 4 were implicated in the innate immune response, and 62 were identified as genes associated with the cellular response to stress (Table S1).

#### 3.2.5. KEGG Enrichment Analysis of Differentially Expressed Genes

**Figure 6:**
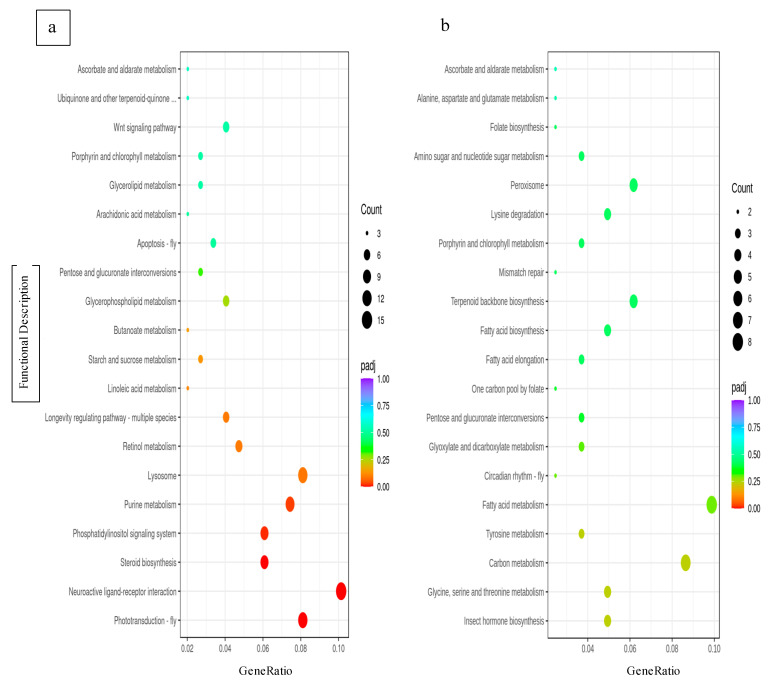
Dot plot of KEGG pathway enrichment analysis of up (**a**) and down (**b**) regulated differentially expressed genes. Gene ratio is the percentage of differentially expressed genes in each term. Diameter of circles represents the relative number of genes within each functional gene identity. Larger diameter represents a greater number of counts, and smaller diameter circles represent lower numbers of counts. Color of the circles represents the adjusted *p*-value (padj) of the gene identities, with a lower *p* value being represented in red and higher *p*-value represented by violet.

Among the upregulated genes identified in the KEGG analysis were components of: the fly apoptosis pathway (53/3195); cytochrome p450 4C1 (37/3195); metabolism associated with cytochrome p450 4C1 (39/3195); insect hormone biosynthesis (32/3195); and apoptosis in multiple species (23/3195) (Figure 6a), where the fractional value is the BgRatio, the ratio of all genes that are attributed to the term. Very few differentially expressed genes were downregulated, and none of the significantly downregulated genes were significantly associated with a known biological pathway (Figure 6b). Of those downregulated genes, many were identified as components of metabolism pathways.

#### 3.2.6. Glycolysis Pathway

**Figure 7:**
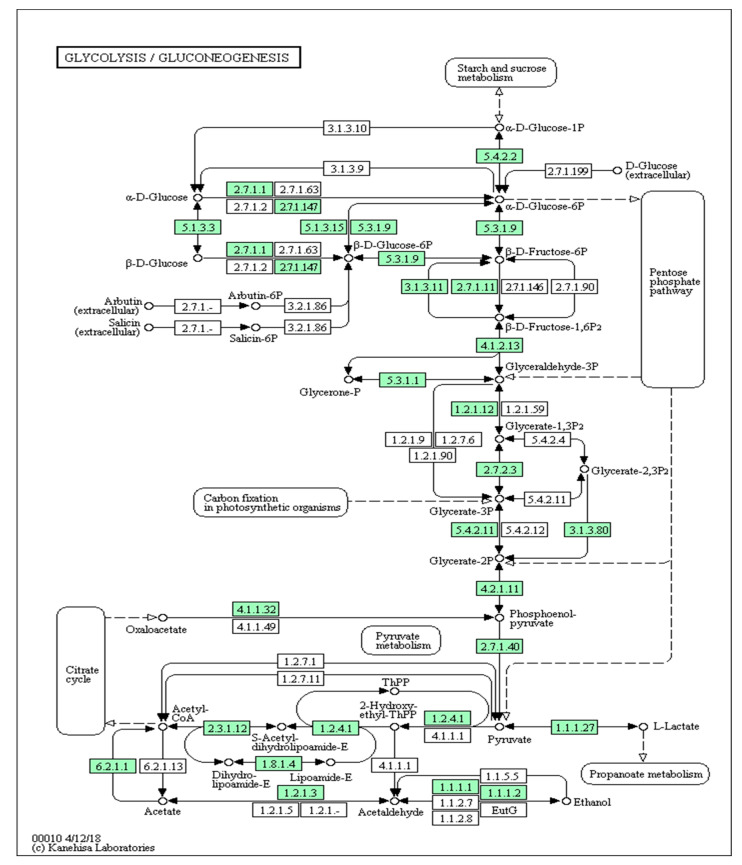
Fire ant glycolysis pathway, where components notated in green were upregulated in infected colonies.

Twenty-four pathway components that are steps of the fire ant glycolysis pathway were upregulated, each of which is notated in green (Figure 7, provided by Novogene Laboratories). Pathway components and function of steps in the glycolysis pathway are as described in Table 2.

## 4. Discussion

Control of the invasive red imported fire ant has been a major goal since its introduction in the early 1900s, and the fire ant pathogen SINV-3 is a potential biological control candidate. In this work, we study the fire ant immune response to SINV-3 using a transcriptome sequencing approach. Most of the differential gene expression seen prior to and following infection was upregulation (Figure 2a); relatively few genes were downregulated between infection statuses (Figure 2b). Many of the transcripts were expressed at detectable levels in both infected and uninfected colonies.

The principal component analysis parses the variation in expression levels. The first PCA1 plots the very high proportion of DGE accounted for by infection status. PCA2 plots the variation among the feral field-caught, SINV-3 uninfected colonies and shows that, after laboratory SINV-3 infection, differential gene expression patterns cluster tightly. Transcripts related to innate immune response to infection status were differentially expressed; among the DEGs were components of the janus kinase (JNK), allatostatin, antimicrobial peptide secretion, cytochrome p450, immunodeficiency (Imd), Toll, apoptosis and glycolysis pathways.

### 4.1. Janus Kinase (JNK)

Only one component of the JNK pathway was differentially expressed. Infected colonies showed upregulation of JNK interacting protein 1, when compared with uninfected colonies (2.497 log2 FoldChange, *p*-value 0.000). This is evidence that the JNK pathway is not significantly implicated in the fire ant immune or cellular repair response to SINV-3 infection. This is important, as the JNK pathway is associated with an exceptionally wide range of cellular, developmental and inflammatory processes in insects [25,26]. Also important is the lack of significantly downregulated JNK pathway components. In other systems, including the small brown planthopper *Laodelphax striatellus*, viral infection appears to induce increased JNK activity, which also appears to increase viral replication and production, whereas JNK suppression decreases viral replication and proliferation [25]. The existence of such a relationship between the fire ant host and its natural enemy SINV-3 remains to be studied, but the data presented is suggestive that no such relationship exists between SINV-3 and the fire ant JNK pathway.

### 4.2. Allatostatin

Allatostatin has been shown to play a major role in development regulation and the secretion of juvenile hormone, but has also been shown to play a role in immune response in the model organism Drosophila. This appears to be the case here as well. In Drosophila, a robust upregulation of allatostatin receptors is seen upon microbial challenge [27]. A correlation is also seen between upregulation in allatostatin and immune activation of antimicrobial peptides (AMPs), including cecropin A1 [27]. In the case of SINV-3 infection, we see both increased expression of allatostatin A2 (1.422 log2FoldChange, *p*-value 0.000) and the allatostatin A receptor (1.307 log2FoldChange, *p*-value 0.000), accompanied by increased expression of antimicrobial peptide genes. This evidence is supportive of the implication of allatostatin and its activation of antimicrobial peptide secretion in the fire ant immune response to SINV-3.

### 4.3. Antimicrobial Peptides (AMPs)

Two genes responsible for production of major royal jelly protein were upregulated in infected ants when compared with their uninfected counterparts (2.100 log2FoldChange, *p*-value 0.000 and 7.970 log2FoldChange, *p*-value 0.000, respectively). Major royal jelly proteins are implicated in a number of functions in the honey bee, including social immunity and larval development. Major royal jelly protein 1 presence provides insights to a potential avenue by which the fire ant may be combatting infection. In the honey bee, nurses have been shown to incorporate fragments of pathogens and dsRNA into the royal jelly for the purpose of eliciting an immune response in colony members who consume it [28,29,30]. Upregulation of major royal jelly proteins are of particular interest to the response landscape of SINV-3, in light of the fact that the fire ant is able to withstand SINV-3 prevalence in the invasive range, and it is possible that the fire ant is using a similar methodology to the honey bee in the clearance of SINV-3. Importantly, all colonies involved in this study succumbed to SINV-3 infection within 10 days post-infection. Similar studies looking at expression of major royal jelly proteins in colonies that survive infection would be worth pursuing.

### 4.4. Cytochrome p450

A number of cytochrome p450 genes were upregulated in colonies post-infection. Cytochrome p450 is a source of major interest in insect detoxification and metabolism of a number of substrates. Roles in insecticide resistance have been established in multiple insect species, and host–plant interactions have also been identified in the insect response to defense molecules secreted by the host plant [31]. The function of cytochrome p450 in insect growth and development is also far reaching. Lesser known is the role of cytochrome p450 function in ants, specifically the ant immune response to the many challenges that plague them. The picture remains ambiguous; when infected with SINV-3, *S. invicta* showed increased expression of eleven cytochrome p450 genes, including three analogs of cytochrome p450 4C1 (3.597 log2FoldChange, *p*-value 0.000; 1.800 log2FoldChange, *p*-value 0.0076; and 1.394 log2FoldChange, *p*-value 0.0018, respectively). The opposite is seen in the case of *S. invicta* parasitism; p450 4C1 is lowly expressed in parasitized fire ant heads, relative to those parasitized by the fire ant decapitating phorid *Pseudacteon curvatus* [32,33]. Cytochrome p450 has been shown to play a role in the stress response in the midgut of the silkworm *Bombyx mori*, and is involved in induction of apoptosis [34]. All of the cytochrome p450 4C1 genes identified for differential gene expression across infection status were upregulated in the infected colonies.

### 4.5. Toll and Immune Deficiency (Imd) Pathways

In eusocial Hymenoptera, the involvement of the immune deficiency (Imd) pathway in defense against viruses is somewhat cryptic, as it is implicated in immune response and in regulation of development [35]. Transmembrane receptor PGRP-LC acts as the principal receptor in initiation of the Imd pathway in systemic infection, or in the localized midgut response in the anterior section of the midgut in localized infection [36,37,38,39], which provides some insight as to its involvement in SINV-3 infection, as SINV-3 has been previously shown to replicate in the midgut of the fire ant [11]. While further work is required to further elucidate the intricacies of Imd involvement in SINV-3 infection, it is likely that it is both implicated directly in immune response and in tissue development following infection.

Upregulation of multiple Toll pathway components was expected, as Toll is a highly conserved pathway involved in immune response and developmental processes [10,40] that is implicated in the viral immune response of other insects [41,42,43,44]. Transcriptome sequencing studies similar to ours in the honey bee have shown that Toll plays a complicated role in the honey bee viral immune response; young bees infected with Israeli Acute Paralysis virus (IAPV) exhibited increased expression of Toll as compared with mature bees that did not [10,16,45]. Similarly, recent work in the Argentine ant *L. humile* also implicated Toll as being central to viral immunity [42]. This trend appears to hold true for the fire ant in response to SINV-3 infection. We saw three genes associated with the Toll and Imd signaling pathways upregulated (Gene_id 105201790, 105197939, 105207936) (Table S1*).* These genes were upregulated exclusively and continuously in all infected colony samples. Further work is required to confirm the implication of Toll and Imd pathway components in the SINV-3 viral immune response, but consistent upregulation of pathway components across infected samples is suggestive of Toll and Imd involvement.

### 4.6. Apoptosis

The role of apoptosis in insect immune response is highly variable. Depending on the system, apoptosis can serve as a survival mechanism of either viral pathogen or host. In some insects, apoptosis can serve as a gatekeeper for prevention of viral proliferation, where destruction of infected cells eliminates virus infected cells and prevents viral replication [46,47]. Conversely, virus-induced apoptosis is a mechanism employed by some entomopathogen systems for proliferation of viral particles by release of pathogens from the dead cells following hijacking of cellular machinery [48,49]. KEGG analysis identified downregulation of components of both the fly apoptosis pathway (Gene_id 105193719, 105201954; Table S1) and of apoptosis pathway components conserved across multiple species (Gene_id 105193719; Table S1). This data is suggestive of one of two scenarios in the SINV-3 infected fire ant; either the fire ant is suppressing apoptosis in order to prevent viral dissemination, or SINV-3 has an inhibitory effect on the fire ant apoptosis pathways in order to maximize SINV-3 production by infected cells. Further work is required to tease apart this relationship, and it is worth noting that the apoptosis pathway components noted were downregulated in all infected colony samples.

### 4.7. Glycolysis

The glycolysis pathway had numerous components upregulated in infected colonies (Figure 7, Table 2). The implications of glycolysis pathway upregulation are highly relevant to immune response vertebrates and are now recently being recognized in invertebrates. Mounting a successful immune response requires the rapid reallocation of energetic resources, and a metabolic shift from fueling normal cellular activity to that of immune response. In the absence of immune challenge, the glycolysis pathway is largely associated with regulation of metabolism at the molecular level, energy storage and tissue development. In the instance of immune challenges, however, the glycolysis pathway facilitates a metabolic “switch” that reallocates energetic resources to immune associated functions [50]. Importantly, such a switch must occur rapidly for production of ATP, as resistance or the mounting of innate immunity requires fast, timely and energetic response.

Metabolic reprogramming due to immune challenge has been described in Drosophila, referred to as the Warburg effect. Under such metabolic switching, immune cells “prefer” glycolysis over oxidative phosphorylation even in the presence of oxygen [33,50] to provide for energetic needs. In short, in the absence of immune challenge, glycolysis is involved in ATP production via the mitochondria. When energetic needs increase during the immune response, cellular metabolism of immune pathway components changes from a state of minimal energetic requirement to high demand. When this occurs, the classical route of ATP synthesis via the mitochondria does not generate energetic resources rapidly enough to mount a successful immune response upon recognition of an antigen. While ATP synthesis via glycolysis generates relatively small amounts of energy, it is still more rapid and productive than oxidative phosphorylation, so immune metabolism preferentially operates using glycolysis, rather than oxidative phosphorylation.

The Warburg effect has been documented in insect transcriptome studies, namely in mosquito, Drosophila and tobacco budworm systems [50,51]. Reallocation of glucose resources from anabolic activity to the production of antimicrobial peptides (AMPs) is among the first steps in the insect immune cascade that serves as both a direct immune regulator on their own as well as an initiator of other immune cascades, including the Toll, Janus kinase (Jnk) and JAK/STAT pathways. The fat body plays two major roles in insect immune response relative to the glycolysis pathway and to energetic resource allocation. When the anabolic switch toward immune response is made, fat body energetic reserves are reallocated to immune pathway components. Antimicrobial peptide secretion is initiated in the fat body. That initiation is supported by the increased expression we saw of the AMPs ataxin, jelleins (specifically major jelly protein), and arrestin in SINV-3 infected fire ants. In sum, upregulation of numerous glycolysis pathway components, paired with increased expression of fat body associated antimicrobial peptides, is evidence of a robust and energetic immune response by the fire ant in response to SINV-3 infection, and supportive of a Warburg effect in the fire ant.

## 5. Concluding Remarks

Control of the red imported fire ant has been a subject of extensive research interest, and the discovery of entomopathogens that infect this invasive pest are gaining traction as biological control agents. Here, we used a transcriptome sequencing approach to identify upregulation of components of immune response pathways implicated in fire ant immunity and SINV-3 infection. In doing so, we found differential expression of several known immune response genes and have thus expanded knowledge about how the fire ant responds to immune challenge.

## Figures and Tables

**Table 1:** Total reads generated with Illumina sequencing and total reads and that mapped to the *S. invicta* reference genome (percentage of total reads).

Sample Identity	Total Reads	Total Reads Mapped to *S. invicta* Genome
Infected colony #1	41059816	36869843 (89.8%)
Infected colony #2	63672854	35140919 (55.19%)
Infected colony #3	43475766	37892796 (87.16%)
Infected colony #4	41430720	37170634 (89.72%)
Infected colony #5	50718212	45360352 (89.44%)
Infected colony #6	52744884	42313142 (80.22%)
Uninfected colony #1	43370224	36954917 (85.21%)
Uninfected colony #2	52867232	46842630 (88.6%)
Uninfected colony #3	50842784	42388449 (83.37%)
Uninfected colony #4	59334108	38106930 (64.22%)
Uninfected colony #5	52962938	44766349 (84.52%)
Uninfected colony #6	37330324	32719501 (87.65%)

**Table 2:** Components of the glycolysis pathway that are upregulated in fire ants following SINV-3 infection, and their function in the glycolysis pathway.

Upregulated Components of the Glycolysis Pathway		
Figure Identification Number	Gene ID	Pathway Component Identification
5.4.2.2	105199081	phosphoglucomutase-2
2.7.1.1	105202325	hexokinase-2
2.7.1.147	105205417	ADP-dependent glucokinase
5.1.3.3	105199419	aldose 1-epimerase isoform X1
5.1.3.15	105207919	glucose-6-phosphate 1-epimerase
5.3.1.9	105206436	glucose-6-phosphate isomerase
3.1.3.11	105198274	fructose-1,6-bisphosphatase 1
2.7.1.11	105204005	ATP-dependent 6-phosphofructokinase isoform X2
4.1.2.13	105198376	fructose-bisphosphate aldolase isoform X1
5.3.1.1	105192814	triosephosphate isomerase
1.2.1.12	105196341	glyceraldehyde-3-phosphate dehydrogenase 2-like
2.7.2.3	105198397	phosphoglycerate kinase
5.4.2.11	105193833	phosphoglycerate mutase 2
3.1.3.80	105194144	multiple inositol polyphosphate phosphatase 1 isoform X1
4.2.1.11	105197049	enolase
4.1.1.32	105192993	phosphoenolpyruvate carboxykinase
2.7.1.40	105201593	pyruvate kinase isoform X1
2.3.1.12	105197159	dihydrolipoyllysine-residue acetyltransferase component of pyruvate dehydrogenase complex, mitochondrial isoform X1
1.2.4.1	105198508	pyruvate dehydrogenase E1 component subunit beta, mitochondrial-like
1.1.1.27	105201857	L-lactate dehydrogenase B chain
6.2.1.1	105199849	acetyl-coenzyme A synthetase
1.8.1.4	105198927	dihydrolipoyl dehydrogenase, mitochondrial
1.2.1.3	105192904	retinal dehydrogenase 1
1.1.1.1	105194743	alcohol dehydrogenase class-3

## Data Availability

Data available to collaborators upon request.

## Supplementary Materials

The following supporting information can be downloaded at: https://www.mdpi.com/article/10.3390/genes14010188/s1, Table S1: Differential gene expression of innate immune response genes consequent to *Solenopsis invicta* Virus-3 infection.
